# Incorporation of Pork Meat and Blood Plasma Proteins into a Cocoa Cream Matrix: Characterization, Comparison of Functional Properties, and In Vitro Simulated Digestion

**DOI:** 10.3390/foods14142547

**Published:** 2025-07-21

**Authors:** Milica Stožinić, Đurđica Ačkar, Branislav Šojić, Tea Sedlar, Ljiljana Popović, Biljana Pajin, Ivana Flanjak, Maja Bulatović, Jovana Petrović, Ivana Nikolić, Ivana Lončarević

**Affiliations:** 1 Faculty of Technology Novi Sad, University of Novi Sad, Bulevar cara Lazara 1, 21000 Novi Sad, Serbia; milica.stozinic@uns.ac.rs (M.S.); sojic@tf.uns.ac.rs (B.Š.); ljiljana04@tf.uns.ac.rs (L.P.); pajinb@tf.uns.ac.rs (B.P.); jovana.petrovic@uns.ac.rs (J.P.); ivananikolic@uns.ac.rs (I.N.); 2 Faculty of Food Technology Osijek, Josip Juraj Strossmayer University of Osijek, Franje Kuhača 20, 31000 Osijek, Croatia; dackar@ptfos.hr (Đ.A.); ivana.flanjak@ptfos.hr (I.F.); 3 Institute of Food Technology, University of Novi Sad, 21000 Novi Sad, Serbia; tea.sedlar@fins.uns.ac.rs; 4 Faculty of Technology and Metallurgy, University of Belgrade, Karnegijeva 4, 11120 Belgrade, Serbia; mbulatovic@tmf.bg.ac.rs

**Keywords:** protein, cocoa cream product, functional properties, amino acid content, in vitro digestibility

## Abstract

Consumer requirements for confectionery products have changed significantly over the past decade. These changes are evident in the growing demand for products that are high in protein but lower in energy content and, as a result, the market for these types of products is expanding. This study compared the chemical composition and functional properties of pork meat protein (MP) and blood plasma protein (BP) and evaluated their incorporation into cocoa cream formulations. Functional properties, such as water-holding capacity (WHC) and oil-holding capacity (OHC), were determined. Essential amino acid profiles were determined using HPLC analysis, and protein digestibility was evaluated both in the native form and after incorporation into the cocoa cream matrix via in vitro enzymatic digestion assays. Additionally, antioxidant activity of the enriched cocoa creams was assessed using the established ABTS assay. Results showed that BP contained a higher proportion of essential amino acids (26.44% of total amino acids), meeting the FAO/WHO recommendations, and exhibited superior digestibility compared to MP. Both proteins demonstrated high WHC and OHC values. The antioxidant potential of BP-enriched cocoa cream further supported its functional benefits. These findings indicate that blood plasma protein is a promising ingredient for enhancing the nutritional and functional quality of cocoa cream products.

## 1. Introduction

Confectionery products, particularly chocolate and cocoa cream spreads, are appreciated and enjoyed by people worldwide. Their distinctive flavor and enticing aroma, attributed to the presence of cocoa, set them apart and contribute to their global popularity [1]. These products are traditionally formulated with high amounts of sugar and fat, while their protein content remains relatively low.

As consumer awareness of nutrition grows, the demand for reformulated confectionery with improved nutritional profiles, particularly higher protein content, is steadily increasing. In recent years, global dietary patterns have shifted toward foods that offer both enjoyment and health benefits. High-protein diets are associated with numerous positive outcomes, including improved satiety, support for healthy aging, weight management, and prevention of chronic conditions, such as obesity and hypertension [2,3,4]. This growing interest in protein-enriched foods has prompted the food industry to innovate and diversify protein sources, especially in products that are not traditionally considered protein-rich, such as sweets and spreads.

Socioeconomic changes, such as economic development and urbanization, are also influencing consumer behavior, particularly in low- and middle-income countries, where the most significant growth in demand for animal-based foods is observed [5]. To meet this increasing demand sustainably, researchers and food producers are exploring not only conventional protein sources but also alternative and underutilized ones. Different animal products (e.g., meat, eggs, and dairy products) are considered excellent sources of high-quality protein, with egg protein often regarded as the benchmark for a complete protein due to its ideal amino acid profile [6,7]. Approximately 30% of the world’s meat consumption consists of pork. In 2013, the highest consumers of pork were Luxembourg and Spain (98.53 and 90.04 kg/per person and 52 kg/per person, respectively), slightly surpassing countries like Malta, The Netherlands, and Portugal [8]. Despite its nutritional advantages, pork protein is associated with high monetary and environmental value [9].

At the same time, the meat processing industry generates substantial quantities of by-products, including bones, internal organs, and blood [8]. These materials, although often discarded or used for low-value purposes, contain high-value compounds that can be recovered and reused, supporting both environmental sustainability and circular economy models [10,11]. Among these by-products, animal blood is especially promising due to its high protein and heme iron content.

Blood plasma, the largest protein-rich fraction of blood, consists mainly of globulins, albumins, and fibrinogen [8]. Due to its unique composition, blood plasma exhibits a wide range of functional characteristics, such as water-binding capacity, foaming, gelation, and emulsifying ability, making it a suitable ingredient for a variety of processed food applications. Pork blood, in particular, is commonly used as a stabilizer, emulsifier, and binder, and it has more recently been explored in different food matrices [12,13,14,15]. Blood plasma is already used in traditional products in Europe and Asia, such as blood puddings, sausages, crackers, and blood curd [16,17]. However, its potential use in sweet food systems, such as confectionery, remains largely unexplored. Given the increasing demand for protein-enriched foods and the need to valorize meat industry by-products, the application of blood plasma protein in products like cocoa cream spreads presents an innovative solution.

Cocoa cream products are marked by their high sugar and fat content, while typically containing low amounts of protein, making them suitable candidates for enrichment with pork-derived proteins. Furthermore, comparing these two protein types in the same matrix allows the evaluation of their nutritional and techno-functional behavior under similar conditions. This study aims to analyze and compare pork proteins derived from different parts of the animal, meat, and blood plasma, assess their functional properties before their incorporation into cocoa cream products at varying concentrations of protein (10%, 12.5%, and 15%), and explore their potential health benefits.

## 2. Materials and Methods

### 2.1. Materials

Materials used for this study included commercially available proteins: pork meat protein (MP) and pork blood plasma protein (BPP), kindly donated by Gombit Ltd. (Inđija, Serbia). Materials used for the preparation of cocoa cream products—cocoa powder, milk, and sugar powder—were generously donated by Pionir Ltd. (Subotica, Serbia), and plant-based fat MKP was donated by Dijamant Ltd. (Zrenjanin, Serbia).

### 2.2. The Chemical Composition of Commercial Proteins

The contents of moisture, fat, protein, ash, and total dietary fiber were determined by following the AOAC method (No. 931.04, No. 963.15, No. 939.02, No. 933.22, and No. 958.23) [18]. The energy value was calculated by multiplying the values of protein and carbohydrates with 17 kJ/100 g, fiber with 8 kJ/100 g, and the fat with 37 kJ/100 g. The content of carbohydrates was calculated using the following formula:

(1) %Carbohydrate=100−(%moisture+%protein+%fat+%ash+%fiber content)

### 2.3. The Determination of Amino Acids Using the HPLC-FLD Method

HPLC analysis was conducted using a Shimadzu HPLC system (Kyoto, Japan) that included a Shimadzu LC-20AD solvent delivery module, a Shimadzu CTO-20AC column oven, a Shimadzu SIL-10AF autosampler, and a Shimadzu RF-20Axs fluorescence detector, all managed through LabSolution Lite software (Release 5.52). The commercially available proteins MP and BP (0.5 g each) were weighed in screw-cap tubes, and 10 mL of HCl (6 M) was added. Afterwards, the tubes were placed in an electric oven at 110 °C for 24 h, cooled, and filtered through Sartorius 389 filter paper into a volumetric flask and diluted up to 25 mL with ultrapure water. Hydrolyzed samples were stored at +4 °C until further analysis. A 120 µL aliquot of the hydrolyzed sample was placed in a glass tube. Then, 150 µL of borate buffer (pH 10.2) was added, and the mixture was vortexed for 30 s. Next, 30 µL of o-phthalaldehyde reagent, 30 µL of 9-fluorenylmethoxycarbonyl chloride reagent, and 1920 µL of ultrapure water were added, with thorough vortexing following the addition of each reagent. Prior to HPLC analysis, the solutions were filtered through a 0.45 µm nylon membrane filter. Separation of fluorescent amino acid derivatives was conducted using a Shimadzu Intersil ODS-3V column (250 × 4.6 mm, 5 µm). The column temperature was maintained at 40 °C. The mobile phase comprised 40 mM sodium phosphate (NaH_2_PO_4_) at pH 7.8 as solvent A, while solvent B consisted of a mixture of acetonitrile, HPLC-grade methanol, and ultrapure water in a ratio of 45:45:10 (*v*:*v*:*v*). The flow rate of the mobile phase was set to 1 mL/min, with the following gradient conditions: starting with 15% solvent B, the percentage of solvent B was linearly increased to 55% over 40 min, which was then maintained for an additional 40 min. Finally, at the 65 min mark, the initial conditions (15% solvent B) were restored. Detection of separated amino acids was performed at an excitation wavelength of 340 nm and an emission wavelength of 450 nm, except for proline, which was detected at excitation and emission wavelengths of 266 nm and 305 nm, respectively. Identification of each amino acid in the sample was achieved by comparing its retention time to that of the corresponding amino acid in a standard solution. Quantification of the identified components was performed using an external calibration method. The results were expressed as milligrams of each amino acid per 100 g of the sample.

### 2.4. The Determination of Functional Properties of Proteins

#### 2.4.1. Protein Solubility (WSI—Water Solubility Index)

The solubility of proteins (MP and BPP) in water was determined according to the method in [19] with certain modifications, as follows: 10% aqueous protein solution (*m*/*v*) was prepared by dispersing proteins in distilled water with stirring on a magnetic stirrer for 1 h, and then it was left to stand in the refrigerator for 24 h. After that, the pH of the solution was adjusted to 2.0, 4.0, 6.0, 8.0, and 10.0 using 1 M NaOH and 1 M HCl. The solution was centrifuged under the following conditions: 10,000× *g* for 15 min. The protein content was determined in the supernatant, as well as the total protein content. Protein water solubility (WSI) was determined using the following formula:

(2) $$WSI(\%)=\frac{protein\;content\;in\;supernatant\;(mg)}{total\;protein\;content\;(mg)}\times 100\%$$

#### 2.4.2. Water-Holding Capacity (WHC)

The water-holding capacity was determined according to the method of Diniz and Martin [20]: 0.25 g of the sample was dissolved in 10 mL of distilled water. After 30 min, the sample was centrifuged (Eppendorf, Hamburg, Germany) for 20 min at 2800× *g*. The water-holding capacity (WHC) was determined by the formula:

(3) $$WHC(\%)=\frac{volume\;of\;supernatant\;(mL)}{initial\;volume\;(mL)}\times 100\%$$

#### 2.4.3. Oil-Binding Capacity (OAC—Oil Absorption Capacity)

The protein sample (0.25 g) was dissolved in extra virgin olive oil (10 mL). The test sample was centrifuged (Eppendorf, Hamburg, Germany) at 2800× *g* for 20 min after 30 min [20]. The oil absorption capacity (OAC) was determined using the formula:

(4) $$OAC(\%)=\frac{volume\;of\;supernatant\;(mL)}{initial\;volume\;(mL)}\times 100\%$$

#### 2.4.4. Foam Production (FC—Foam Capacity)

Foaming ability and foam stability were determined by the method of Naczk, Diosady, and Rubin [21]. The tested protein solution (10% *w*/*v*) was vigorously mixed with a homogenizer at 10,000 rpm for 1 min (T-25 Ultraturax, IKA, Shanghai, China). Foam capacity (FC) was determined as follows:

(5) $$FC(\%)=\frac{\left(A-B\right)}{A}\times 100\%$$

In which:

A—the volume of the test sample measured after 1 min of vigorous mixing (mL),
B—sample volume before mixing (mL).

#### 2.4.5. Emulsifying Properties

The emulsifying properties (activity and stability index) of a 10% solution of the tested protein (*m*/*v*) were determined according to the method of Pearce and Kinsella [22]. First, the protein solution was diluted 100 times, and then it was mixed with olive oil in a ratio of 2:1 and vigorously vortexed. The absorbance of the sample was measured at 500 nm.

The emulsion activity index (EAI) was determined using the formula:



(6) $$EAI(m^{2}/g)=\frac{\left(2\times 2.303\times A_{0}\times N\right)}{\left(L\times c\times \varphi \times 10,000\right)}$$

The emulsion stability index (ESI) was determined as follows: the absorbance of the tested protein solution was read immediately after vortexing and after 24 h in the refrigerator. The ESI was calculated according to the following formula:

(7) $$ESI(min)=\frac{A_{0}}{∆A}\times t$$

where:

A_0_—absorbance of emulsion immediately after vortexing,
N—dilution factor (100),
L—the thickness of the cuvette used for the spectrophotometer, which was 1 cm,
c—protein concentration in the sample (g/mL),
φ—oil content in the emulsion,
ΔA—change in absorbance during storage time in the refrigerator,
t—time interval of storage in the refrigerator (24 h).

### 2.5. The Preparation of Cocoa Cream Products

Cocoa cream products were prepared in a ball mill at a temperature of 40 °C during 1.5 h at the maximum speed of the mixer. The batch weighed 3 kg. A control sample (C) was obtained from powdered sugar (50%), cocoa powder (10%), vegetable fat (30%), milk powder (10%), sunflower lecithin (0.5%), and vanillin (0.04%) for the control sample. Additionally, enriched cocoa cream products were prepared in the same manner as the control, with the incorporation of 10%, 12.5%, and 15% of pork meat protein (MPC10, MPC12.5, and MPC15) and pork blood plasma (BPC10, BPC12.5, and BPC15). The goal was to develop products that can be labeled as a source of protein.

### 2.6. In Vitro Digestion

In vitro digestion of protein isolates was conducted using a modified method from Mineskus et al. [23]. The procedure involved measuring 1 g of both protein and enriched cocoa cream products. The digestion process took place at 37 °C, starting with a first phase at pH 3 for 1 h using 0.04 g of pepsin, followed by a second phase at pH 7 for 2 h using 0.04 g of pancreatine (Megazym, Bray, Ireland). The enzyme-to-substrate ratio used was 1/25. For the cocoa cream products, α-amylase (Megazym, Ireland) was added at the very beginning of the experiment during the pH 7 phase to mimic the conditions found in saliva for 2 min at 37 °C. After this initial step, the remaining digestion conditions for both protein and cocoa cream products were identical. Once the digestion was complete, the mixture was centrifuged at 14,500 rpm for 10 min, and the supernatant was collected and kept in the freezer for further analysis.

### 2.7. The Degree of Hydrolysis (DH)

The degree of hydrolysis of protein and cocoa cream products was determined using the method by Popović et al. [24]. A mixture of 0.5 mL of supernatant and 0.5 mL of 0.44 mol/L TCA was prepared and incubated at 4 °C for 30 min before centrifugation at 14,500 rpm for 5 min. The Lowry method [25] was then employed to determine the protein content in the TCA-soluble fraction and total proteins in the supernatant. The protein concentration in enriched cocoa cream products was calculated by dividing the calibration curve value by a corrective factor: 0.1 for BPC10 and MPC10, 0.125 for BPC12.5 and MPC12.5, and 0.15 for BPC15 and MPC15, according to the added concentration of protein in the cocoa cream sample. The degree of hydrolysis (DH) was calculated accordingly:

(8) $$\%DH=\frac{TCA\;proteins}{Total\;proteins}\times 100\%$$

### 2.8. Total Phenol Content

The total phenol content was determined using the method developed by Singleton et al. [26]. To prepare the reaction mixture, we combined 0.1 mL of the appropriate extract dilution, 7.9 mL of distilled water, 0.5 mL of Folin–Ciocalteu reagent, and 1.5 mL of 20% Na_2_CO_3_. In this context, extract refers to the digest obtained from in vitro digestion. The Folin–Ciocalteu reagent was prepared by mixing one part of 2M Folin–Ciocalteu with two parts of distilled water. After the incubation for 1 h at room temperature, the absorbance of the tested sample was measured at a wavelength of 750 nm. The total phenol content in the analyzed samples was expressed as mg of gallic acid equivalents per g of sample (mg GAE/g).

### 2.9. Determination of ABTS Radical Scavenging Activity

Here, 30 µL of obtained digest was added to 3 mL of diluted solution of cation radical ABTS. For the assay, the ABTS stock solution was created by combining 2.45 mmol/L of potassium persulfate with 7 mmol/L of ABTS (2,2′-azino-bis(3-ethylbenzothiazoline-6-sulfonic acid) diammonium salt). Following 16 h of incubation at room temperature, the absorbance of the ABTS stock solution was adjusted to 0.70 ± 0.02 using acetate buffer at pH 3.6. The blank was a mixture of 30 µL of acetate buffer (pH 3.6) combined with ABTS solution. The absorbance was measured for the sample and control after 10 min at 734 nm. ABTS radical scavenging activity was determined using the following formula [27]:

(9) $$\%AA=\frac{Ablank-Asample}{Ablank}\times 100$$

### 2.10. Statistical Analysis

The collected data were analyzed employing a statistical approach referred to as ANOVA. A one-way ANOVA was conducted to compare the means, with simultaneous comparisons performed using Duncan’s test at a significance threshold of 0.05, utilizing Statistica 13.0 software. Each experiment was replicated three times to ensure reliability of the results.

## 3. Results and Discussion

### 3.1. Chemical Composition of Proteins

The pork-derived proteins, including meat protein and purified blood plasma proteins, were subjected to analysis, and the corresponding results are summarized in Table 1. The results indicate significant differences (*p* ≤ 0.05) between the samples of pork protein in terms of protein, ash, moisture, fat, fiber, carbohydrate, and energy content. The MP sample had a higher content of protein (90.67 ± 0.6223), implying higher purity. The BP sample was fat-free, since the blood plasma contains low levels of fats [28]. Additionally, it was observed that the moisture level in the BP sample was 2.8-fold higher compared to the MP sample.

Regarding the content of ash, higher values were observed in the BP sample, with a 2.8-fold increase compared to the MP sample. This can be explained by the presence of minerals, like calcium, sodium, magnesium, potassium, and iron, which are abundant in the porcine blood plasma [29].

### 3.2. Amino Acid Profile

Proteins are formed from amino acids, which serve as the building blocks and play essential structural and metabolic roles in humans and animals [30]. Amino acids can be classified according to their role in the human organism. The first and most important group consists of essential amino acids, which the body cannot synthesize. It includes valine, threonine, methionine, leucine, lysine, isoleucine, histidine, tryptophane, and phenylalanine. The second group, conditionally non-essential, contains arginine, tyrosine, and cysteine. Lastly, the third group, which contains non-essential amino acids, is comprised of glycine, serine, glutamic and aspartic acid, alanine, glutamine, asparagine, and proline [31]. The amino acid composition of meat protein and blood plasma protein is presented in Table 2.

According to the results, essential amino acids comprised 15.11% of the total amino acid content in the MP sample and 26.44% in the BP sample, showing a significant difference among the samples (*p* ≤ 0.05). The major amino acids detected in this study were cystine, glycine, and methionine. The most dominant essential amino acid in the MP sample was threonine (34.21 mg/100 g), responsible for maintaining gut homeostasis, and it has a crucial role in regulating nutritional metabolism and synthesizing macromolecules [32]. Methionine, a precursor of cysteine, creatine, and carnitine, plays a role in the recycling of sulfur, which is afterward used in energy-consuming reactions [33]. In the BP sample, methionine was the most dominant essential amino acid (94.35 mg/100 g). Regarding the non-essential amino acids, the highest amount was observed for the cystine amino acid (328.15 and 718.22 for MP and BP, respectively). In a study conducted by Almeida et al. [34], amino acid composition was determined in dehydrated porcine plasma. The results of this study indicated a significant difference (*p ≤* 0.05) between this protein and the BP sample, with levels of threonine, histidine, and methionine in the BP sample being 1.5-, 3-, and 10-fold higher, respectively. The amounts of remaining essential amino acids in the BP sample were lower than in the protein from the study in [34]. This phenomenon can be explained by many factors, such as different processing conditions (heat treatment) or the diet of an animal. Furthermore, if the Maillard reaction occurs, it can lead to degradation, mostly of lysine [35,36,37]. According to the FAO/WHO report [38] on the essential amino acid requirements for adult humans, the current estimate is 277 mg of essential amino acids per gram of protein. In the analyzed protein samples, the MP sample contained 90.05 mg/g, which does not meet the recommended requirements. In contrast, the BP sample had 311.75 mg/g of protein, which satisfies the FAO/WHO guidelines.

These results imply that the BP protein has a higher level of amino acids, particularly essential regarding the content of essential amino acids that are important for the optimal health of humans. Therefore, it could be considered a potential source of protein of animal origin.

### 3.3. Functional Properties of Proteins

Proteins designed for incorporation into food matrices should exhibit specific functional properties relevant to the intended application, enabling efficient production while preserving the integrity of the final product. The functional properties of proteins in food refer to the physicochemical characteristics that determine their behavior and interactions in food systems [39]. Functional properties, such as solubility (WSI), water- and oil-holding capacity (WHC and OHC, respectively), foaming capacity (FC), and emulsifying activity index and stability (EAI and ES, respectively), of the analyzed proteins are presented in Table 3.

The two different types of proteins, extracted from distinct parts of pork, exhibited significant differences (*p* ≤ 0.05) in their functional properties. The results indicate that the BP had a high solubility level of 95.7%, whereas the MP demonstrated low solubility at 13.4%. This can be explained by the difference in the protein structure, mainly because BP consists of water-soluble fractions—albumins and globulins [40]. In contrast, MP is comprised of sarcoplasmic proteins (water soluble), myofibrillar proteins (soluble in high-salt solutions), and stromal proteins, which are mostly insoluble [41]. Alvarez et al. [40] reported that the solubility range in isolated pork plasma ranged from 80% to 100%, while Choi et al. [42] confirmed the low solubility of meat protein derived from pork. These findings show that different kinds of proteins, based on their solubility, have limited applications in different food matrices. Moreover, both samples exhibited a high percentage of water-holding capacity (WHC), above 80%, which is undesirable in cocoa cream products. The high WHC can possibly lead to the absorption of water from the environment, negatively affecting the texture, causing oil migration, and reducing the shelf life of the products with high oil content [43]. This parameter is influenced by particle size, as smaller particles tend to exhibit higher WHC values due to their increased ability to trap water within the voids between them [44,45,46]. According to literature data, the typical WHC range for MP falls between 70% and 76% [47,48,49]. Based on the results obtained for the oil-holding capacity (OHC), it appears that the earlier concerns related to the high WHC may be mitigated, indicating that the cocoa cream samples produced could maintain stability. The emulsifying capacity and stability were also evaluated, with the BP sample showing an emulsifying capacity that is 1.32 times higher than that of the MP sample, demonstrating that the BP has the potential to be incorporated into various products whose production involves emulsification processes, such as mayonnaise, pâtés, and sausages [12].

Future studies could focus on examining the effects of added protein on particle size distribution, color, rheological properties, and the shelf life of enriched cocoa cream products to determine the behavior of proteins in produced samples.

### 3.4. Determination of Degree Hydrolysis (DH)

**Figure 1:**
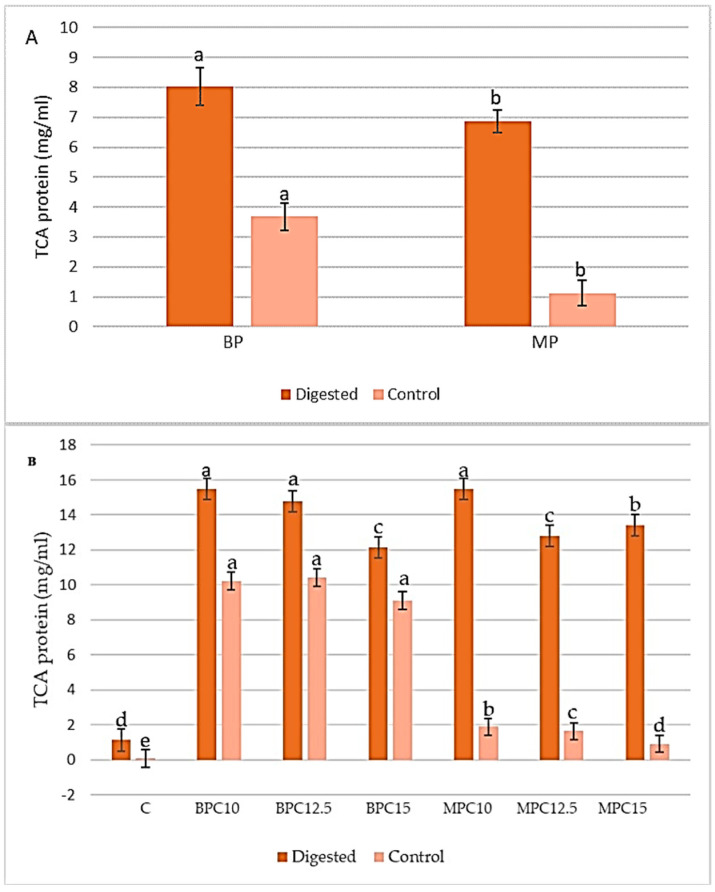
Protein content in TCA-soluble fraction before and after digestion. (**A**) Protein BP and MP and (**B**) cocoa cream samples enriched with 10, 12.5, and 15% of protein. Values followed by the same letter for the same treatment are not significantly different (*p* ≥ 0.05).

Comprehensive understanding of protein characteristics, especially digestibility, is essential for assessing their bioaccessibility and overall nutritional quality [50]. Therefore, the preliminary phase involved the comprehensive preparation and treatment of raw materials to effectively eliminate anti-nutritional factors. This process was succeeded by the extraction and isolation of proteins under optimized parameters to ensure maximal yield and functionality. However, this study employed commercially available proteins, thereby bypassing the extraction and isolation stages entirely. Subsequently, in vitro digestion was conducted, after which the determination of the soluble protein fraction (TCA protein) was performed to accurately assess the bioaccessibility and digestibility of the isolated proteins [50]. The obtained results, shown in Figure 1, indicate that a significant difference (*p* ≤ 0.05) was present among the digested samples and their controls, indicating effective protein degradation.

The highest TCA protein concentration was present in the cocoa sample enriched with 10% of added protein, either BP or MP (15.474 mg/mL). The lowest value was in the control sample (1.129 mg/mL) since the source of protein in this sample was only milk and cocoa powder. Certain peptides, originally present in the cocoa, exhibited resistance to in vitro digestion, particularly those with a Phe or Leu residue at the C-terminal, showing a significant decrease in bioaccessibility [51].

**Figure 2:**
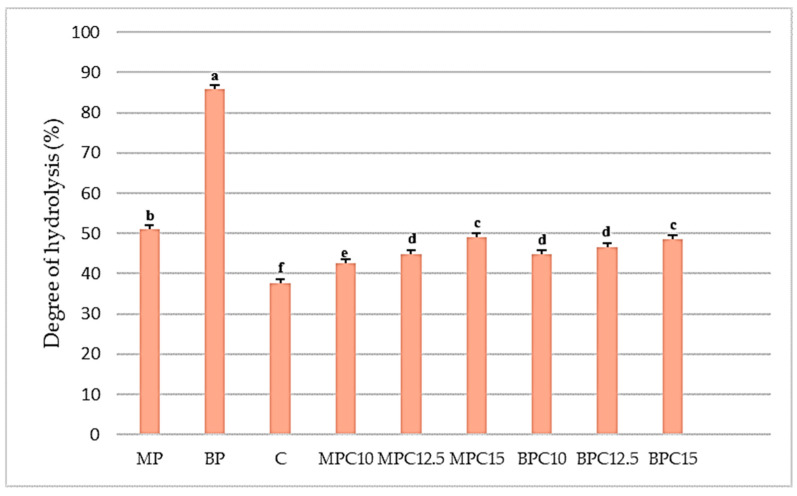
Degree of hydrolysis in protein (BP and MP) and cocoa cream samples enriched with 10, 12.5, and 15% of protein. Values followed by the same letter are not significantly different (*p* ≥ 0.05).

Samples with more than 10% of protein showed a decrease in TCA protein content, probably due to the interactions in the matrix and the complexity of the system, which included cocoa, sugar, and milk particles, besides the fat and emulsifier. The same trend was present after the calculation of the degree of hydrolysis (DH). The results are presented in Figure 2.

After comparing the results, the highest value for the degree of hydrolysis was detected in the BP protein sample (85.81%). However, after incorporating the BP into the cocoa cream matrix, the degree of hydrolysis decreased with the increase of protein in cocoa samples. The same trend was observed for the MP protein and corresponding samples. The control sample exhibited the lowest value (37.51%). Reduced digestibility in enriched cocoa cream products may be due to polyphenol compounds found in cocoa powder, which can form conjugates with proteins, lowering their bioaccessibility and digestibility, as supported by several studies [49,52]. Many studies have reported the presence of biological functions in hydrolyzed peptides derived from pork meat and by-products, such as antioxidant, antihypertensive, and antimicrobial activity [53,54,55].

### 3.5. Total Phenol Content (TF)

**Figure 3:**
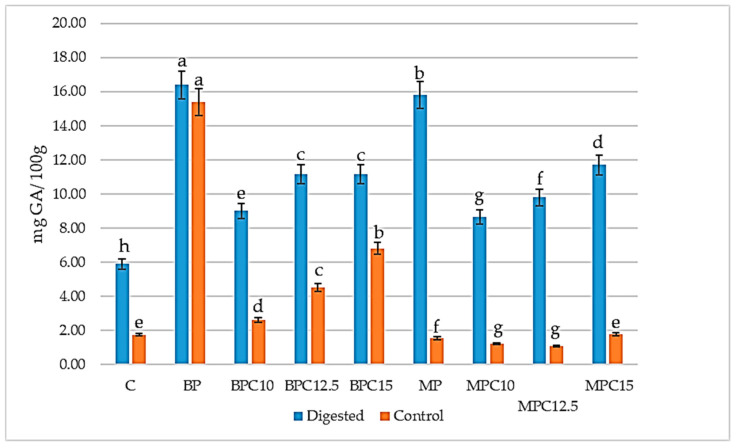
Total phenol content in protein (BP and MP) and cocoa cream samples—control and samples enriched with 10, 12.5, and 15% of protein. Values followed by the same letter for the same treatment are not significantly different (*p* ≥ 0.05).

Polyphenols are bioactive compounds, found mostly in plant-based foods and recognized for their role in preventing cardiovascular disease, osteoporosis, cancer, and diabetes mellitus [56,57]. The results of total phenol content are presented in Figure 3. Ozdal et al. [58] reported on the interactions between the phenol compounds and proteins, both reversible and irreversible. Conjugation refers to the interaction between various compounds, resulting in structural modifications due to the formation of specific chemical bonds between the reactive groups of the substances involved. When proteins interact with polyphenols, they form “protein–polyphenol conjugates”. In essence, proteins and polyphenols can link together through either non-covalent bonds, such as hydrophobic, ionic, and hydrogen bonds, or covalent bonds, whether enzymatic or non-enzymatic [31,59]. These interactions can lead to changes in digestibility and physicochemical properties of proteins [60]. Since phenols are plant compounds, they are usually not present in animal products [61]. The presence of phenolic compounds in protein isolates derived from porcine blood plasma and meat may result from the animal’s diet, especially when it includes polyphenol-rich feed ingredients (e.g., pomace, soybean meal, cereals, legumes, or fruit and vegetable by-products). After absorption in the small intestine, polyphenols enter the portal circulation and reach the liver, where they are mainly present as glucuronides and methylated glucuronides. These polar metabolites can undergo further modifications in cells and are primarily excreted by the kidneys. There are two main metabolic pathways: one leads to excretion of polyphenol metabolites in the urine, and the other involves their transport to the colon. A significant portion of ingested polyphenols (80–90%) reaches the colon either unabsorbed or after being absorbed, metabolized, and then secreted back into the gut via bile or membrane transporters [61,62,63,64]. Several studies investigated and confirmed the presence of phenolic compounds from an animal diet [65,66,67] and their interactions with proteins, indicating the presence of covalent bonding with meat and plasma proteins [68,69,70]. The results showed that the highest TF content was observed for the BP sample, both digested and control (16.39 ± 0.09 and 15.39 ± 0.12, respectively). For digested samples, the lowest value was observed in the C sample—1.75 mg GAE/100 g, and for control the lowest was for MPC10 and MPC12.5—1.22 and 1.09, respectively. After analyzing the results, it was noticeable that the higher enrichment of cocoa cream products with both proteins increased the content of phenolic material. In the C sample, the source of phenolic compounds was cocoa powder, rich in polyphenols [71].

### 3.6. ABTS Radical Scavenging Activity

**Figure 4:**
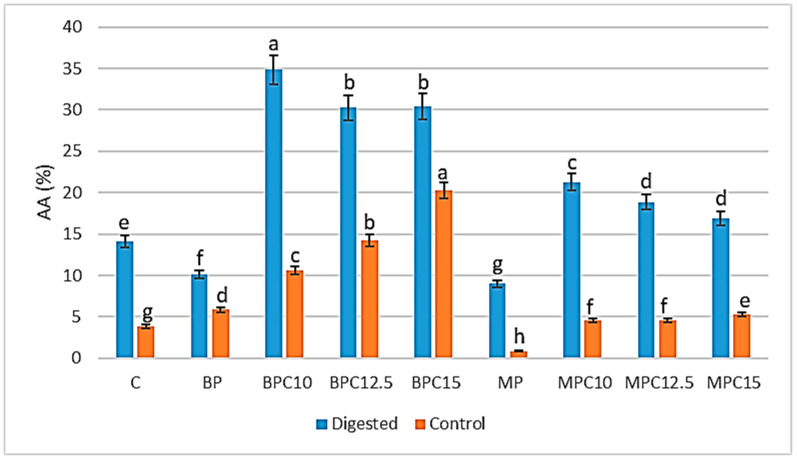
ABTS radical scavenging activity (AA%) in protein (BP and MP) and cocoa cream samples—control and samples enriched with 10, 12.5, and 15% of protein. Values followed by the same letter for the same treatment are not significantly different (*p* ≥ 0.05).

Determination of antioxidant activity in digested and control samples was carried out by the ABTS method, and the results are presented in Figure 4. The antioxidative capacity of the hydrolysates was determined by their amino acid sequence [72]. Additionally, many studies reported the effect of the degree of hydrolysis on the antioxidative activity [72,73,74].

The results of the ABTS assay showed that the radical scavenging activity of proteins and enriched cocoa cream products was enhanced in digested samples compared to the non-digested counterpart. The highest values in digested samples were observed for the BPC10 sample (34.87%), and in control samples, the highest value was present in MPC15 (20.28%). For the digested samples, BPC12.5 and BPC15 did not show significant differences (*p* ≥ 0.05), as well as MPC12.5 and MPC15. In control samples, only MPC10 and MPC12.5 did not show any significant difference. During the in vitro digestion, BP and MP in their native form, or incorporated into the matrix, were hydrolyzed into peptides, which exhibited greater antioxidative activity. This conclusion was confirmed by several studies [75,76]. The greater antioxidant capacity observed in the digests can be attributed to the enzymatic release of bioactive peptides during the in vitro digestion process. These peptides, which are encrypted within the primary structure of native proteins, become bioactive only after proteolytic fractionation [77,78]. Hydrolysis enhances antioxidant potential by exposing or generating specific amino acid sequences with radical-scavenging, metal-chelating, and reducing properties [79,80]. The antioxidant activity of these peptides is strongly influenced by their amino acid composition, sequence, and structure. Peptides containing residues, such as tyrosine, tryptophan, histidine, cysteine, methionine, and lysine, contribute significantly due to their ability to donate hydrogen atoms or electrons to stabilize free radicals [80,81,82]. Aromatic amino acids (e.g., Tyr and Trp) are especially effective due to their capacity to donate protons, enhancing radical-scavenging potential [83]. Histidine-containing peptides also display antioxidant activity via hydrogen donation, metal ion chelation, and lipid peroxyl radical trapping, attributed to the imidazole ring [83,84,85]. The SH group in cysteine can directly interact with radicals, providing an independent antioxidant mechanism [84]. In addition, the position of these amino acids within the peptide sequence plays a critical role in determining their bioactivity [83]. Thus, the elevated antioxidant capacity in digested samples likely results from a combination of mechanisms, including direct radical scavenging, metal chelation, and synergistic effects of multiple small peptides generated during enzymatic hydrolysis. These effects are not merely additive but integrative, suggesting that the total antioxidant activity reflects the collective behavior of all released peptides [82].

## 4. Conclusions

This study concluded that BP protein, classified as a by-product and waste of the meat industry, has significant potential as an innovative source of protein for food products. The amino acid profile of BP met the essential amino acid requirements of the FAO/WHO guidelines for adult humans. Additionally, the results of HD demonstrated good bioaccessibility of the BP sample, both in its native form and when incorporated into a cocoa cream product. The functional properties of the BP and MP samples suggest their diverse applications in food matrices. The authors assume that the diet of the animal from which the proteins were derived influenced the presence and content of phenol compounds, responsible for antioxidant and anti-inflammatory properties. Hydrolyzed proteins exhibited higher bioactive values compared to the control. Future research can focus on determining the oxidative stability of produced cocoa cream samples. Additionally, sensory evaluation, rheological properties, color assessment, hardness, and thermal characteristics should be examined to better assess the potential industrial applications of BP. This study highlighted the significant potential of BP protein, a by-product of the meat industry, as an innovative protein source for food products, emphasizing its relevance to the food industry. With the increasing demand for sustainable and alternative protein sources, the findings support the idea of utilizing waste materials, such as BP, to create value-added food products. The amino acid profile of BP aligned with the FAO/WHO guidelines for essential amino acids in adult humans, making it a viable option for enhancing nutritional quality in various food applications. Moreover, the demonstrated good bioaccessibility of BP, both in its native form and when integrated into products like cocoa cream, showcased its adaptability and potential for incorporation into diverse food matrices. The industry can benefit from exploring BP’s functional properties, as well as its bioactive values compared to hydrolyzed proteins.

However, limitations of this study include a lack of information regarding the sustainability and technological parameters crucial to industry relevance, e.g., particle size distribution, rheological properties, and oxidative stability. A few studies indicated that the implementation of blood plasma in food matrices, such as frankfurter sausages, emulsion-type sausages, and sponge cakes, did not significantly impact the sensory attributes compared to their counterparts [86,87,88], indicating the consumer acceptance of BP in different food matrices. Additionally, one of this study’s limitations is the lack of sensory analysis, which will be conducted in future studies. By focusing on aspects such as oxidative stability, sensory attributes, rheological properties, color assessment, and thermal characteristics, future research can provide insights that facilitate the development of innovative confectionery products and other food items. This not only aligns with current trends in sustainability but also offers manufacturers the opportunity to reduce waste while meeting consumer demands for healthy and functional foods. Overall, the study underscores the importance of leveraging by-products from the meat industry to create new market opportunities and foster sustainable practices in food production.

## Figures and Tables

**Table 1:** Chemical composition of protein, carbohydrate, moisture, fat, fiber, and ash.

	MP	BP
Proteins	90.67 ± 0.6223 ^a^	86.85 ± 0.5869 ^b^
Carbohydrate	0 ^a^	1.59 ± 0.1 ^b^
Fiber	2.23 ± 0.071 ^a^	0.9 ± 0.106 ^b^
Fat	0.9 ± 0.02 ^a^	0 ^b^
Moisture	3.87 ± 0.034 ^a^	4.12 ± 0.0216 ^b^
Ash	2.33 ± 0.0141 ^a^	6.54 ± 0.0071 ^b^
Energy (kJ/100 g)	1541.39	1510.68

Values expressed as g/100 g, except for energy (mean values). Values followed by the same letter within the same row are not significantly different (*p* ≥ 0.05).

**Table 2:** Amino acid profile in mg/100 g.

Amino Acid (mg/100 g)	MP	BP
Aspartic acid	2.19 ± 0.38 ^a^	6.91 ± 0.14 ^b^
Glutamic acid	9.09 ± 1.11 ^a^	11.10 ± 0.01 ^b^
Serine	2.95 ± 0.77 ^a^	10.12 ± 0.53 ^a^
Histidine	2.28 ± 0.58 ^a^	69.73 ± 3.65 ^b^
Glycine	118.42 ± 14.19 ^a^	66.93 ± 3.98 ^a^
Threonine	34.21 ± 1.79 ^a^	70.60 ± 8.17 ^b^
Arginine	27.63 ± 3.86 ^a^	18.59 ± 3.09 ^b^
Alanine	13.67 ± 1.63 ^a^	27.56 ± 2.24 ^b^
Tyrosine	3.34 ± 1.08 ^a^	7.73 ± 1.34 ^b^
Cystine	328.15 ± 36.63 ^a^	718.22 ± 54.67 ^b^
Valine	4.86 ± 0.78 ^a^	22.45 ± 0.96 ^b^
Methionine	25.95 ± 2.25 ^a^	94.35 ± 1.04 ^b^
Tryptophane	0.38 ± 0.11 ^a^	0.97 ± 0.01 ^b^
Phenylalanine	5.37 ± 0.64 ^a^	15.70 ± 1.23 ^b^
Isoleucine	6.27 ± 0.77 ^a^	2.34 ± 0.35 ^b^
Leucine	11.22 ± 1.35 ^a^	35.53 ± 1.66 ^b^
Lysine	n.d. ^1,a^	0.26 ± 0.00 ^b^
Proline	n.d. ^a^	n.d. ^a^

**^1^** n.d.—not detected. Values expressed as mg/100 g (mean values). Values followed by the same letter within the same row are not significantly different (*p* ≥ 0.05).

**Table 3:** Functional properties of MP and BP.

	MP	BP
WSI (%)	13.4 ± 0.3464 ^a^	95.7 ± 0.4582 ^b^
WHC (%)	83.4 ± 1.0583 ^a^	100 ± 1.2135 ^b^
OHC (%)	93.6 ± 1.1358 ^a^	85.8 ± 1.3868 ^b^
FC (%)	-	72.3 ± 0.8185 ^b^
EAI (m^2^/g)	183 ± 0.8185 ^a^	243 ± 1.6093 ^b^
ES (min)	28 ± 0.5 ^a^	88 ± 1.5620 ^b^

Values expressed as mean values. Values followed by the same letter within the same row are not significantly different (*p* ≥ 0.05).

## Data Availability

The original contributions presented in this study are included in the article. Further inquiries can be directed to the corresponding author.

## Abbreviations

The following abbreviations are used in this manuscript:

MP	Meat protein
BP	Blood plasma
WSI	Water solubility index
WHC	Water-holding capacity
OHC	Oil-holding capacity
FC	Foaming capacity
EC	Emulsion activity index
ES	Emulsion stability
DH	Degree of hydrolysis
